# CLINICAL AND NUTRITIONAL ASPECTS IN OBESE WOMEN DURING THE FIRST YEAR AFTER ROUX-EN-Y GASTRIC BYPASS

**DOI:** 10.1590/S0102-6720201500S100016

**Published:** 2015-12

**Authors:** Tiago Dália dos SANTOS, Maria Goretti Pessoa de Araújo BURGOS, Maria da Conceição Chaves de LEMOS, Poliana Coelho CABRAL

**Affiliations:** Hospital das Clínicas, Federal University of Pernambuco, Recife, PE, Brazil

**Keywords:** Obesity, Roux-en-Y gastric bypass, Weight loss, Women

## Abstract

**Background:**

Clinic care for morbid obesity is not very effective. Bariatric surgery is being considered the best way of intervention for this kind of obesity.

***Aim*:**

Evaluate the clinical and nutritional evolution during the first year of obese women submitted to Roux-en-Y gastric bypass.

***Method*:**

Retrospective series non-concurrent with 61 women. The variables were weight, BMI, weight loss percentage, loss of excessive weight percentage, waist circumference, hip circumference, lipid profile, daily use of supplements, practice of physical exercise, occurrence of sickness, nausea, constipation, diarrhea, asthenia, alopecia, dry skin, cramps and brittle nails.

***Results*:**

They presented significant weight and IMC reduction as well as improvement in their lipid profile, in all consultations. After one year they presented 36,6% loss of the initial weight and 75% loss of excessive weight. The waist circumference also presented a considerable reduction on all the moments, decreasing from 122,1±13,4 cm to 94,1±10,6 cm. Regarding the intercurrences, the most frequent were alopecia, asthenia, dry skin and cramps.

***Conclusion*:**

The Roux-en-Y gastric bypass was effective in promoting and maintenance weight loss during the period of the first postoperative year.

## Abstract (pt)

***Racional*:**

O tratamento clínico para a obesidade mórbida é pouco eficaz. A cirurgia bariátrica tem sido considerada a melhor forma de intervenção para esse tipo de obesidade.

***Objetivo*:**

Avaliar a evolução clínico-nutricional durante o primeiro ano de mulheres obesas submetidas ao bypass gástrico em Y-de-Roux.

***Métodos*:**

Estudo retrospectivo não concorrente do tipo série de casos com 61 mulheres. As variáveis analisadas foram peso, IMC, percentual de perda de peso, percentual de perda do excesso de peso, circunferência da cintura, circunferência do quadril, perfil lipídico, uso diário de polivitamínico-mineral, prática de exercício físico, presença de vômitos, náuseas, constipação, diarréia, astenia, alopecia, pele seca, câimbras e unhas quebradiças.

As pacientes apresentaram redução significativa do peso e IMC bem como melhora do perfil lipídico, em todas as consultas avaliadas. Ao final de um ano apresentaram 36,6% de perda do peso inicial e 75% de perda do excesso de peso. A circunferência da cintura também apresentou redução significante em todos os momentos, diminuindo de 122,1±13,4 cm para 94,1±10,6 cm. No que se refere às intercorrências, as mais frequentes foram vômitos, alopecia, astenia e unhas quebradiças.

***Conclusão*:**

O bypass gástrico em Y-de-Roux foi efetivo na promoção e manutenção da perda de peso no primeiro ano pós-operatório.

## INTRODUCTION

Obesity is defined as excessive accumulation of body fat that can impair health^18^. It is a chronic disease caused by multiple factors, resulting from the interaction of genes, environment, lifestyle and emotional factors. Reaches epidemic proportions worldwide and its prevalence has shown rapid growth in all age groups in Brazil^15^.

In Brazil, according to the survey Risk Factors Surveillance for Chronic Diseases and Protection through telephone interviews (VIGITEL 2013) the frequency of obese adults was 17.5%. In males, the obesity rate in the range 18-24 years and 25-34 years of age, was 8.1% and 16.4% respectively, reaching the highest prevalence in the range 55-64 years (22.3 %), declining after 65 (16.5%). Among women, the frequency of obesity tended to increase with age up to 54 years (18 to 24 years: 4.4%; 25 to 34 years: 13.7%; 55-64 years old: 25.9%), also having reduction of the prevalence in the range after 65 (22.9%)^14^.

 Morbid obesity is linked to considerable risk to health, with development of several metabolic disorders and associated diseases such as type 2 diabetes mellitus, hypertension, dyslipidemia, among others. Given this fact, bariatric surgery has emerged as the most effective treatment for patients with surgical indication^21^. It has been identified as the only treatment to achieve weight loss (PP) appropriate and durable. Surgical treatment for obesity comprises a set of surgical techniques that aims at quick and effective weight loss, concomitantly associated with the treatment of diseases associated and/or aggravated by morbid obesity^17^.

Thus, this study aims to evaluate the clinical and nutritional status of patients undergoing Roux-en Y gastric bypass (RYGB) during the first postoperative year. 

## METHODS

The study was approved by the Ethics Committee of the Federal University of Pernambuco (registration CEP/CCS/UFPE No. 639049). It was retrospective not competitor series of cases with obese women, inserted in the Bariatric Surgery Program, Hospital das Clínicas, Federal University of Pernambuco, Recife, PE, Brazil, underwent RYGB and accompanied the nutrition clinic in Gastroplasty in the period 2010-2013.

Data were collected from medical records, charts and ambulatory monitoring through a collection form developed for this research. The collected variables refer to the following stages of treatment: immediately before surgery, 1, 4, 7 and 12 months after surgery.

The evaluated anthropometric data were: weight (kg), body mass index, weight loss percentage (%PP) loss percentage of overweight (%EWL), waist circumference (WC) and hip circumference (HC).

 The %PP was calculated on the pre-surgical weight and current weight (considered as weight on 1^st^, 7^th^, 12^th^ and 24^th^ month of operation) from the formula: (pre-surgical weight - current weight)/presurgical weight x 100). The %EWL was calculated by the "PP postoperative (after the 1^st^, 7^th^, 12^th^ and 24^th^ month of operation) x 100/preoperative weight - ideal weight", in which the ideal weight was calculated from the formula "height (m)²x25 ".

Were evaluated the lipid profile of patients from the results of determination of serum levels of total cholesterol, HDL-cholesterol, LDL-cholesterol and triglycerides. Data were collected from routine exams requested by outpatient professionals. Values: total cholesterol, 0 - 2000 mg/dl, HDL-cholesterol, 32-72 mg/dl, LDL-cholesterol, 0-115 mg/dl and triglycerides 0-200 mg/dl.

 Were also evaluated the use of vitamin-mineral supplements prescribed by a nutritionist, regular physical exercise and the presence of the following complications: vomiting, nausea, constipation, diarrhea, asthenia, alopecia, dry skin, cramps and brittle nails in all queries. These reports were computed as reported by the patient at the time of consultation.

The patients with the medical record and/or outpatient follow-up form with incomplete data regarding anthropometric variables were excluded, those who have undergone any surgical procedure within 12 months after gastroplasty and who used hormone therapy.

Data were organized in a database with further processing and analysis in the Statistical Package for the Sciences (SPSS), version 13.0 (SPSS Inc., Chicago, IL, USA). To verify associations between variables were used the Pearson's chi-square and the normality of the data evaluated by the Kolmogorov-Smirnov test. Except for variable %PEP, all other normal distribution and were described as mean and standard deviations and 95% confidence interval. In such cases were used ANOVA for repeated measures followed by post-hoc Bonferroni (5% significance level). The variable %PEP data were presented as median and percentiles 25 and 75, by using the Friedman test with post-hoc Wilcoxon.

## RESULTS

Were admitted to intervention 93 women with a mean age of 40.1±11.8 years. Of these, 61 were followed for 12 months, with loss of the sample of 34.4% over the period of follow-up due to lack of essential data on outpatient records. Of these, 42 had complete data for evaluation of CC, QC and lipid profile.

Table 1 shows the evolution of anthropometric parameters of the patients, the first consultation before the operation, and the other four subsequent queries. Before the operation the women had on average 115,2+/-19,9 kg; BMI 46,0+/-6,1kg/m^2^; 56,9+/-17,7kg overweight (EP); 122,1+/-13,4 cm CC; and CQ 136,2+/-14,1cm.

On postoperative evaluation anthropometric, except for QC, was observed progressive and statistically significant reduction at each time evaluated (Table 1). However, only 50.8% reached the goal which was 10% of PP in the first month, but at the end of follow-up loss was around 33.0%, which was considered sufficient.

The BMI had a 41,3+/-5,8 kg/m^2^ for 30,8+/-4,8kg/m^2^ between the first and the last query. It also observed that 85.24% of the women obtained BMI<35 kg/m^2^ after one year varying from 19,11-34,83 ​​kg/m^2^.

About PEP%, 93.4% of the study population showed losses ranging from 50.5 to 144.2% at the end of follow-up, reaching the expected target of 50-75%; 6.6% did not reach the goal.

 Among them, 96.7% presented %PP provided the goal that was between 20-30%, 93.44% achieved the target for %PEP, which was advocated between 50-75% and BMI should be less than 35 kg/m^2^ after one year, reaching 85.2% after one year.

The CC had considerable reduction in all measurements during the first year, between 114,4 cm, average first month, 94,1 cm after a year of gastroplasty. The CQ showed no statistically significant reduction in any of the four stages.

Some patients had regained weight at the end of follow-up, evidenced in the last visit, corresponding to 4.9% of the population, only three varying the gain between 700-1600 g.

Table 2 shows the evolution of the parameters evaluated lipid profile. No significant differences were shown in any of the components during follow-up. However, there was a trend of improved blood lipid profile of patients, reduction of total cholesterol, LDL-cholesterol and triglycerides and increased HDL-cholesterol.

Table 3 shows the results on the frequency of complications related to nutrient deficiency, bowel habits and food intake. Cramps and alopecia proved evident from the fourth month remaining until the seventh numerically significant values ​​compared to 1 and 12 months.

Patients who reported episodes of asthenia showed more in the fourth postoperative month, 86.9% (n=53) compared to the other three times. Dry skin complaints shown in the first and twelfth month, 23% (n=14) and 9.8% (n=6), respectively.

*Qui-quadrado de Pearson

## DISCUSSION

The study consisted of women, most of the population, which characterizes the patients treated at the Nutrition Clinic in Gastroplasty, hospital of the Federal University of Pernambuco. The average age of the group approached the age group with the highest incidence of morbid obesity of the Brazilian female population^21^. Studies point to the predominance of women. Study analyzed 469 monitoring reports of which 83% were women and 16.8% men^13^. It is known that the woman weight control has strong aesthetic motivation and presents important emotional components in relation to food. Another factor is that obesity predisposes the onset of breast cancer, endometrial cancer, polycystic ovaries and infertility^2^. Such factors may explain the increased demand for female health services for bariatric surgery.

In the present study, was observed that the patients had satisfactory and progressive PP during the annual monitoring. All the parameters used for evaluation of postoperative PP (PP, %PP and %EWL) showed efficient weight loss in the population. Novais et al (2010)^17^ in a study of 141 women, also noted significant PP in the 12 months postoperatively, showing the biggest loss up to six months and its stabilization between the first and second year of operation.

**TABELA 1:** - Evolução de parâmetros antropométricos em mulheres submetidas ao bypass gástrico em Y-de-Roux

Variables	Initial X±DP (IC 95%)	1^st^ appointment X±DP (IC 95%)**	2^nd^ appointment X±DP (IC 95%)	3^rd^ appointment X±DP (IC 95%)	4^th^ Consulta X±DP (IC 95%)	p*
Weight (Kg) * (n=61)	115,2±19,9 (110,5-120,6)^a^	103,4±17,8(99,2-108,7)^b^	91,6±15,9(87,7-96,5)^c^	83,1±15,7(79,5-87,8)^d^	76,9±12,8 (73,5-81,5)^e^	0,000
BMI (Kg/m^2)^* (n=61)	46,0±6,1(44,2-47,8)^a^	41,3±5,8 (39,7-43,1)^b^	36,6±5,5 (35,0-38,3)^c^	32,3±5,1 (31,8-34,8)^d^	30,8±4,8 (29,4-32,2)^e^	0,000
Excess weight(Kg)* (n=61)	56,9±17,7 (52,1-61,7)^a^	43,7±16,5(39,2-48,2)^b^	40,0±14,1(27,1-34,8)^c^	21,9±12,8(18,4-25,3)^d^	15,2±6,8(11,9-18,4)^e^	0,000
Weight loss(Kg)* (n=61)	-	11,6±2,6(10,9-12,4)^a^	23,4±5,4 (21,8-25,1)^b^	31,9±7,3 (29,7-34,1)^c^	38,1±10,2 (35,0-41,1)^d^	0,000
Percentage of weight loss^¨¨^ (%) (n=61)	-	10,0 (8,7-11,9)^¨a^	21,5 (17,8-24,1)^¨b^	29,0 (24,6-33,0)^¨c^	36,6 (28,8-43,7)^¨d^	0,000
Percentage of excess weight loss^¨^ (%)* (n=61)	-	23,6±7,7 (21,3-25,9)^a^	46,9±13,7 (42,8-51,0)^b^	63,5±17,4 (58,4-68,7)^c^	75,0±19,8 (69,1-80,9)^d^	0,000
Waist circumference (cm)* (n=42)	122,1±13,4 (114,00-130,2)^a^	114,4±9,8 (108,5-120,4)^b^	104,9±11,8 (97,7-111,9)^c^	96,5±11,5 (89,6-103,5)^d^	94,1±10,6 (87,7-100,5)^e^	0,000
Hip circumference (cm)* (n=42)	136,2±14,1 (130,6-147,7)	128,9±14,7 (119,9-137,8)	119,6±12,8 (111,9-127,3)	115,8±14,8 (106,9-124,8)	110,4±13,5 (102,2-118,5)	0,385

* ANOVA for repeated measures with post hoc Bonferroni; ** Mean ± standard deviation (95% confidence interval) **.

Median (percentiles 25 -75); Friedman test, with post hoc Wilcoxon. Different letters show statistically significant differences at 5%.

**TABELA 2:** - Evolução de parâmetros do perfil lipídico em mulheres submetidas ao bypass gástrico em Y-de-Roux

Variables	1^st^ appointment X±DP (IC 95%)**	2^nd^ appointment X±DP (IC 95%)	3^rd^ appointment X±DP (IC 95%)	4^th^ Consulta X±DP (IC 95%)	p*
total cholesterol (mg/dl)* (n=42)	182,9±49,6 (153,5-211,6)	173,1±27,5 (157,1-188,9)	176,6±33,0 (157,6-195,7)	168,0±27,5 (152,2-183,9)	1,000
HDL (mg/dl)^¨¨^ (n=42)	36,0 (32,0-40,0)^¨^	43,6 (36,0-49)^¨^	46,0 (41,0-52,8)^¨^	50,0 (44,0-60,2)^¨^	0,635
LDL (mg/dl) (n=42)	121,1±23,6 (99,3-142,9)	110,3±21,0 (90,9-129,8)	109,0±18,5 (91,9-126,1)	91,2±39,1 (55,1-127,3)	0,516
triglycerides (mg/dl) (n=42)	138,0±49,1 (105,1-171,1)	113,8±41,9 (85,6-141,9)	108,7±41,4 (80,9-136,6)	103,1±37,8 (77,6-128,5)	0,701

* ANOVA for repeated measures with post hoc Bonferroni; ** mean ± standard deviation (95% confidence interval) **;

Median (percentiles 25 -75); Friedman test with post hoc Wilcoxon. Different letters show statistically significant differences at 5%.

**TABELA 3:** - Frequência do uso de suplementação vitamínico-mineral, prática de exercício físico e intercorrência em mulheres submetidas ao bypass gástrico em Y-de-Roux

Variáveis	1^st^ appointment	2^nd^ appointment	3^rd^ appointment	4^th^ appointment	p ^*^				
	n	%	n	%	n	%	n	%	
Physical exercise									0,202
Yes	-	-	11	18,0	13	21,3	18	29,5	
No	61	100,0	50	82	48	78,7	43	70,5	
Use of multivitamin-mineral									0,303
Yes	42	68,9	38	62,3	40	65,6	48	78,7	
No	19	31,1	23	37,7	21	34,4	13	21,3	
vomiting									1,000
Yes	14	23,0	17,0	27,0	14	23	13	21,3	
No	47	77,0	44	72,1	47	77	48	78,7	
nausea									1,000
Yes	01	1,6	01	1,6	02	3,3	02	3,3	
No	60	98,4	60	98,4	59	96,7	59	96,7	
Cold									0,663
Yes	15	24,6	07	11,5	11	18	12	19,7	
No	46	75,4	54	88,5	50	82	49	80,3	
diarrhea									0,679
Yes	04	6,6	03	4,9	01	1,6	02	3,3	
No	57	93,4	58	95,1	60	98,4	59	96,7	
asthenia									0,000
Yes	01^a^	1,6	53^b^	86,9	02^a^	3,3	01^a^	1,6	
No	60	98,4	08	13,1	59	96,7	60	98,4	
alopecia									0,000
im	11^a^	18,0	33^b^	54,1	27^c^	44,7	16^a^	26,2	
No	50	82,0	28	45,9	34	55,3	45	73,8	
Dry skin									0,001
Yes	14^a^	23,0	01^b^	1,6	02^b^	3,3	06^a,b^	9,8	
No	47	77,0	60	98,4	59	96,7	55	90,2	
cramps									0,000
Yes	04^a^	6,6	53^b^	86,9	40^c^	80,3	01^a^	1,6	
No	57	93,4	08	13,1	21	19,7	60	98,4	
brittle nails									0,362
Yes	04	6,6	07	11,5	08	13,1	08	8,9	
No	57	93,4	54	88,5	53	86,9	53	91,1	

*Pearson's chi-square

As for BMI and %EWL, Bastos et al. (2013)^4^identified the parameters showed positive results in the evaluation of PP in 114 patients. After a year they had BMI=29.1±4.1 kg/m^2^ and %EWL=66.2±14.9%, as mean±standard deviation.

 Pedrosa et al. (2009)^21^, followed 205 men and women in different periods of postoperative period (6, 12, 18, 24 months). They showed gradual reduction in weight and BMI, with statistical differences at all stages, achieving lower weight and BMI and higher %PP 18-24 months. Santos et al. (2006)^24^ reported that in evaluating the postoperative period of 48 patients, observed gradual reduction of weight and BMI, with statistical difference at all stages (less than six months, 6-11 months and 12-24 months), reaching the period of 12-24 months the lowest weight (82.90± 5.95) and BMI (31.91±5.85) and higher %PP (33.93±2.91).

About %PEP, RYGB associated with the appropriate monitoring by the multidisciplinary team takes the average loss of 75% of pre-surgical EP in the course of a year; however, weight loss over 50% of previous EP at the time of the operation is already considered a good result^8^. The criteria used for classifying the success of the operation (%PEP≥50) it was found that 84% of women achieved it^17^; these findings in this study where the goal that was achieved by 93.44% at the end of the first year.

Studies reveal that significant PP is present at all time points, with reduction from the 12^th^ month of postoperative follow-up. Patients followed showed the expected improvement of anthropometric parameters of evaluation after RYGB.

Ferraz et al. (2003)^7 ^refer %PP as another form of analysis of the PP quality where: excellent, corresponds to loss>35%; good loss 25-34%; poor, loss of 15-24%, and surgical failure loss<15% at the end of a year. There are also indications of surgical success through BMI, and BMI<30 kg/m^2^ as excellent result after a year, between 30-35 kg/m^2^ good result and >35 kg/m^2^ failure^6^. On average, the studied patients achieved the above goals that reveal the surgical success.

Weight regained found here, about 4.9% (n=3) is considered low, but not expected in the first year; PP occurs at accelerated rate in the begining, reducing the rate thereafter. In Sjostromet al (2004)^25^ PP at six months was 33% going to 38% at 12 months. Later there was progressive reduction which continued up to the 10^th^ year of operation. This fact may explain the presence of some patients have presented reduction in PP. Other factors such as the state of hydration and the measurement method can influence the quantification of weight between consultations.

The CC has shown favorable reduction at each visit, so associated with weight loss. Ayoubet al (2011) found reductions between times for CC, both men and women, showing progressive decline over time (up to six months after surgery).

The RYGB operates as follows in the great PP postoperative: 1) limitation of food intake by reducing gastric volume, associated with decreased secretion of ghrelin produced in the stomach, since it is responsible for increasing hunger and intake food; 2) intestinal bypass the proximal portion providing the arrival of quickly nutrients in the distal intestine, where they are present the highest peptide concentrations YY and glucagon stimulating the release of such hormones that act of inhibiting, reducing appetite and thus decreasing food intake; 3) itself decrease the absorptive area provided by intestinal derivation^11^.

Laboratory evaluation of the lipid profile did not differ in all evaluated moments; however, it was observed trend in the reduction of the average values ​​of these biochemical components. It is known that before the operation the patients were taking lipid-lowering drugs and that after the suspension of its use. Julveet al (2014)^10^ showed favorable changes in cholesterol and triglyceride levels in obese patients after one year of surgery. Concomitantly levels were also reduced VLDL-C LDL-C, finding no significant results for HDL-C.

Adiponectin is a hormone that has the function of regulating blood glucose and catabolism of fatty acids. Acts on the lipid profile, regardless, when associated with insulin resistance syndrome features for PP program. Current findings reported that adiponectin is reduced in obese women and increases after the PP induced by RYGB. May suggest then that the present PP is associated with decreased insulin resistance, increased plasma concentrations of adiponectin and improved interaction with lipid profile^9^.

In this study were reported by patients any clinical manifestations such as alopecia, which is verified by Moreira et al (2010)^17^, who found the highest percentage of patients who cited this occurred 90 days after surgery. It has been suggested that the onset of alopecia may result mainly of zinc deficiency and protein malnutrition. However, there are reports that iron deficiency, selenium and copper may cause such phenomenon^23^. In the same study there was no significant results on the complaints of diarrhea, constipation and brittle nails, these were not observed in this study.

In Pavedello et al (2009)^22^, 84.4% of the population had normal bowel habits after surgery, with increased frequency after the operation.

The physical exercise was not identified as significant according to statistical analysis; however, there is reduced habit in the studied population, with an average 70.5% who did not practice physical exercise after 12 months. Also Provedello et al (2009)^22^ shows only 36.4% of patients achieving exercise after 7-18 months postoperatively. Basto et al (2013)^4^ found about 53.1% of those with two or more years after surgery who practiced exercise regularly. In the same study, 73.4% of the sample used the vitamin-mineral supplement regularly, like this one found at every visit frequency greater regular use, ranging from 62.3 to 78.7% when taken into account four times.

Moraes et al (2015)^16^ describe that in a study with women with complaints of alopecia and asthenia proved be significant in postoperative RYGB. This study noted that complaints regarding asthenia showed up in larger amounts in the fourth query monitoring in this study.

Insufficient consumption of iron rich foods in the long-term can lead to the development of iron deficiency anemia, manifested fatigue/ asthenia, irritability, weakness, brittle nails, can lead to more serious consequences if left untreated. Leiro and Melendez-Araújo (2014)^12^ found that in relation to dietary micronutrients, the entire sample showed inadequate iron intake (n=36), all women undergoing RYGB after one year.

The low consumption of iron rich foods associated with slow digestion caused these foods is described as frequent among patients undergoing RYGB. These foods are also high in protein and fiber, nutrients that require time to be digested and delay gastric emptying may cause satiety. With reduced stomach volume due to the surgical technique, this feeling tends to exacerbate. The decrease in production of gastric juice (especially hydrochloric, critical in iron bioavailability optimization reducing the ferric form for ferrous) and the characteristic intestinal bypass this surgical technique, which isolates the portion of the duodenum and the early jejunum - absorption sites this nutrient - are other factors that may contribute to the development of iron deficiency^5^. Unfortunately this study did not evaluate the dietary intake of patients.

Complaints about episodes of cramps were most cited between 4 and 7 months of follow-up. Thiamine, vitamin B and magnesium deficiency may be related to them. Peripheral neuropathy is one of the clinical manifestations of these nutritional disorders, and also characterized by motor and sensory changes^5^^,^ 26, usually manifested as pain, numbness and loss of reflexes. Other symptoms are: numbness in the toes, burning feet, cramps in the calves and lower limb pain^20^. The legs are more affected than the upper limbs, which may explain the high frequency of cramps observed in the study.

It is known that here the patients were not diagnosed with peripheral neuropathy or other neurological disorder resulting from the micronutrient deficiency. However, these signs reported in queries can be explained by the high prevalence of nutritional deficiencies presented by patients undergoing gastroplasty. A retrospective study found a prevalence of 16% of peripheral neuropathy in bariatric surgery^5^. The rapid PP was associated with low serum vitamin B12, thiamine and folic acid^1^.

Complaints related to dry skin are possibly associated with low fluid intake of patients. Valezi et al (2008)^27^ evaluated water intake on 91 women and only 10 (11%) consumed more than 1000 ml per day, most referred intake was approximately 500-1000 ml/day. The reduced intake of water is explained by the reduced gastric volume, but also to poor eating habits that remain after the operation. Therefore, low daily fluid intake becomes factor that predisposes dehydrated skin complaint. Zinc deficiency can also cause drying or breakage of hair and dry skin, which can also be caused by the deficit of vitamin A.

A set of factors involved in post-surgical weight loss is what determines the outcome of the operation on body weight in the short- and long-term, hence the importance of knowing the pattern of weight loss and factors related to it. It must also take into account the monitoring results of the operation, especially in the long-term, that is proven to maintain the benefits of the procedure^17^.

## CONCLUSION

RYGB was effective in the promotion and maintenance of the PP in the period of the first postoperative year.
